# Evolution and physiology of neural oxygen sensing

**DOI:** 10.3389/fphys.2014.00302

**Published:** 2014-08-12

**Authors:** Kauê M. Costa, Daniela Accorsi-Mendonça, Davi J. A. Moraes, Benedito H. Machado

**Affiliations:** Laboratory of Autonomic and Respiratory Control, Department of Physiology, School of Medicine of Ribeirão Preto, University of São PauloRibeirão Preto, Brazil

**Keywords:** oxygen sensing, hypoxia, peripheral chemoreflex, brainstem, autonomic and respiratory control

## Abstract

Major evolutionary trends in animal physiology have been heavily influenced by atmospheric O_2_ levels. Amongst other important factors, the increase in atmospheric O_2_ which occurred in the Pre-Cambrian and the development of aerobic respiration beckoned the evolution of animal organ systems that were dedicated to the absorption and transportation of O_2_, e.g., the respiratory and cardiovascular systems of vertebrates. Global variations of O_2_ levels in post-Cambrian periods have also been correlated with evolutionary changes in animal physiology, especially cardiorespiratory function. Oxygen transportation systems are, in our view, ultimately controlled by the brain related mechanisms, which senses changes in O_2_ availability and regulates autonomic and respiratory responses that ensure the survival of the organism in the face of hypoxic challenges. In vertebrates, the major sensorial system for oxygen sensing and responding to hypoxia is the peripheral chemoreflex neuronal pathways, which includes the oxygen chemosensitive glomus cells and several brainstem regions involved in the autonomic regulation of the cardiovascular system and respiratory control. In this review we discuss the concept that regulating O_2_ homeostasis was one of the primordial roles of the nervous system. We also review the physiology of the peripheral chemoreflex, focusing on the integrative repercussions of chemoreflex activation and the evolutionary importance of this system, which is essential for the survival of complex organisms such as vertebrates. The contribution of hypoxia and peripheral chemoreflex for the development of diseases associated to the cardiovascular and respiratory systems is also discussed in an evolutionary context.

## Introduction

Deprivation of oxygen is one of the most intense physiological challenges for most living organisms. Any human that has suffered from chronic lung disease, been exposed to high altitudes or, for any reason, experienced the sensation of asphyxia knows how dramatic this insult can be. For humans and many other mammals, lack of oxygen for only a few minutes results in rapid cell degradation and, ultimately, death. Not surprisingly, all living beings on Earth have evolved physiological mechanisms that allow these complex multicellular systems to respond to hypoxic situations, preserving cellular integrity to the limit of the organism's capacity.

Included in this repertoire of survival mechanisms are the hypoxia-triggered chemosensitive cells, which, coupled to terminals of afferent neural pathways to the central nervous system (CNS), are in charge of sensing oxygen concentration in the arterial bloodstream and translating this chemical readout into neural information. In the case of low oxygen pressure in the arterial blood, it is activated in order to produce autonomic, respiratory and behavioral responses aimed at protecting the organism's life. In this review, we focus on the evolutionary importance of oxygen sensing and transportation mechanisms, with an emphasis on the vertebrate peripheral chemoreflex. We review the teleonomic function of this mechanism in light of the importance of oxygen for the integrity of complex life and the pivotal role of the nervous system in coordinating oxygen homeostasis. We also discuss the importance of the peripheral chemoreflex in certain pathological conditions and the perspectives for future research on this subject, an important issue for respiratory, cardiovascular and integrative physiologists alike.

## Oxygen: the molecule of complex life

In order to understand the importance of neural oxygen sensing systems, it is mandatory to look at the natural history of this molecule and its relationship with terrestrial life. From a biological point of view, oxygen is the most important of the atmospheric gasses, due both to its unique properties and the role it came to play in the function of the planet's biota. Oxygen *per se* is a rather peculiar element: it is the second most electronegative element, behind fluorine, which makes it an optimal electron acceptor (Allred and Rochow, 1958); it is also the most abundant element on the Earth's crust by mass and the third most abundant element in the universe after hydrogen and helium (Dole, 1965). However, while today a substantial fraction of the Earth's oxygen is located in the atmosphere, the early terrestrial atmosphere was practically anaerobic (Dole, 1965; Nisbet and Sleep, 2001). Levels of all atmospheric gasses, oxygen included, have varied widely over the course of Earth's 4.5 billion year history (Taylor and McElwain, 2010) and concomitantly with major changes in geochemical cycles, these variations have produced profound effects on all of the planet's life forms (Nisbet and Sleep, 2001; Raymond and Segre, 2006; Berner et al., 2007; Koch and Britton, 2007; Harrison et al., 2010; Taylor and McElwain, 2010).

The geochemical analysis of the Earth's geological strata and the study of the fossil record (Berner et al., 2007) reveal an interesting bidirectional relationship: just as oxygen levels came to drive the evolution of living organisms, biological processes heavily influenced the levels of oxygen in the atmosphere (Raymond and Segre, 2006; Berner et al., 2007; Harrison et al., 2010; Taylor and McElwain, 2010). Current evidence shows that the oxidation of the Earth's atmosphere began after early life forms developed oxygenic photosynthesis, i.e., the process of converting CO_2_ into organic compounds using the energy of sunlight and H_2_O as a reductant (Nisbet and Sleep, 2001; Olson, 2006; Buick, 2008). In this process, for each molecule of CO_2_ consumed in the formation of more complex molecules, primarily sugars, a molecule of O_2_ is released to the atmosphere as a byproduct (Olson, 2006; Buick, 2008).

**Figure 1:**
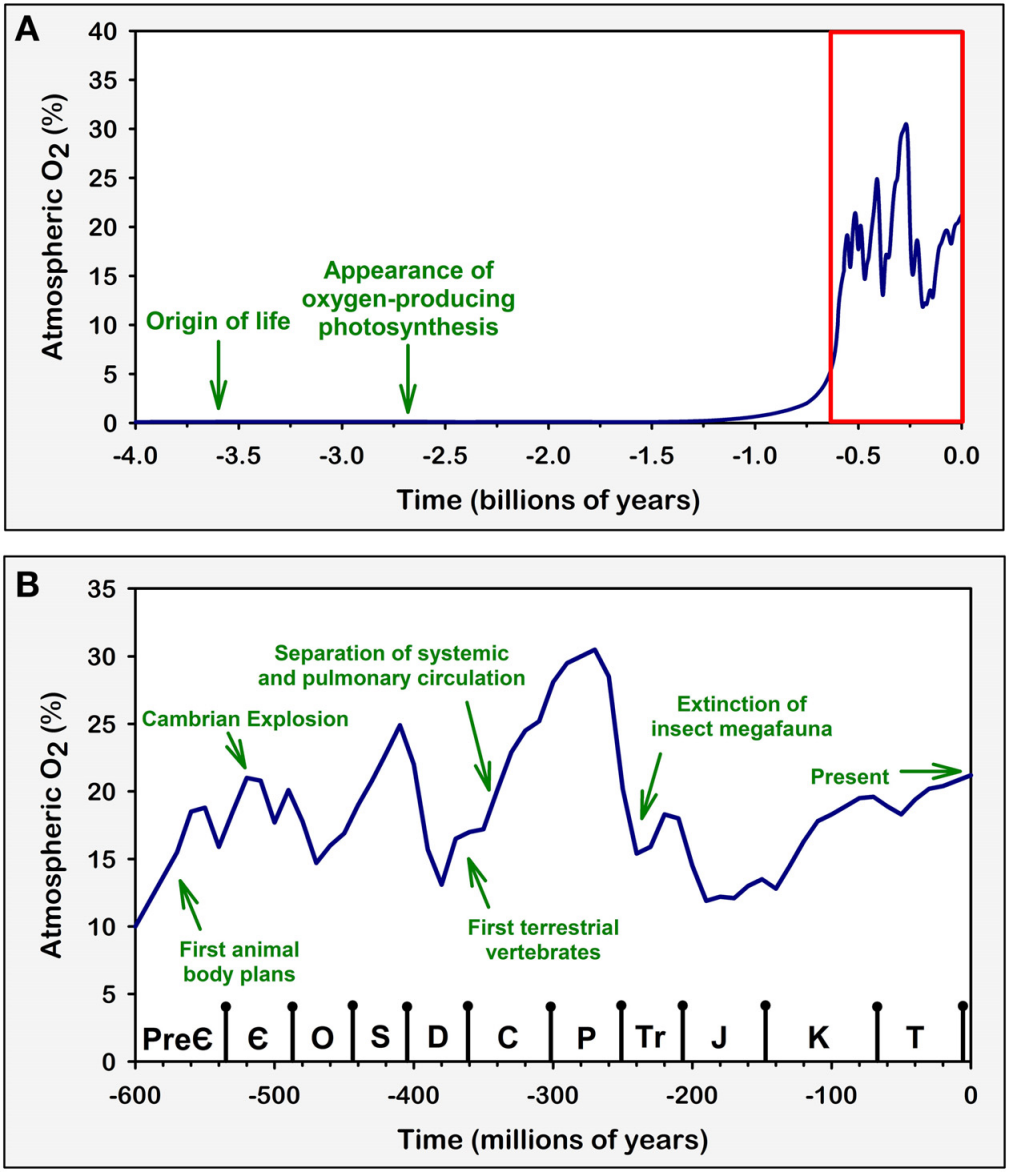
**Variations in atmospheric oxygen concentrations over geological time**. Green arrows and texts represent major evolutionary and geological events that are believed to be in direct connection with atmospheric O_2_ concentration (Graham et al., 1995, 1997; Dudley, 1998; Bishopric, 2005; Raymond and Segre, 2006; Fisher and Burggren, 2007; Koch and Britton, 2007; Harrison et al., 2010; Taylor and McElwain, 2010). Similar comparisons have been discussed previously (Koch and Britton, 2007). Arrows are positioned in time based on approximations derived from geological evidence. Atmospheric O_2_ levels were estimated and plotted based on available geochemical studies (Berner, 2006; Olson, 2006; Raymond and Segre, 2006; Berner et al., 2007; Taylor and McElwain, 2010). **(A)** Atmospheric O_2_ concentration across geological time. This representation includes the periods over which life first appeared on Earth and the evolution of oxygenic photosynthesis, which was responsible for the subsequent rise in atmospheric O_2_. **(B)** A zoom in on the region highlighted in red on **(A)**. This expanded view includes the time span ranging from the Ediacaran period of the late Pre-Cambrian to the present Quaternary period (not named in graph) of the Cenozoic Era. During these periods, atmospheric O_2_ concentration varied greatly; these changes influenced the evolutionary trends of animal life, including the development of the cardiovascular and respiratory systems in vertebrates. Symbols on the bottom axis represent the geological Periods according to the 2009 Geologic Time Scale (Walker and Geissman, 2009). Pre-Є, Pre-Cambrian; Є, Cambrian; O, Ordovician; S, Silurian; D, Devonian; C, Carboniferous; P, Permian; Tr, Triassic; J, Jurassic; K, Cretaceous; T, Tertiary.

Over time, the gradual release of O_2_ by photosynthetic organisms produced a massive increase of atmospheric oxygen levels in the Proterozoic Eon of the Pre-Cambrian period (~2500 to ~540 million years ago), from ~0 to 10% in less than 1 billion years (Raymond and Segre, 2006; Berner et al., 2007; Harrison et al., 2010) (Figure 1A). This oxidation of the atmosphere represented a major challenge for many early anaerobic life forms due to the highly toxic effect that oxygen and reactive oxygen species (ROS) have on living cells (Raymond and Segre, 2006; Monaghan et al., 2009). Consequently, some studies suggest that there was a huge selective pressure that favored the survival of organisms that tolerated oxygen-induced toxicity (Raymond and Segre, 2006). This factor is likely to have contributed to the appearance of organisms that could not only survive in the presence of oxygen but actually had cellular mechanisms that controlled oxidative processes to such an extent that they could use them to effectively generate useful energy (Raymond and Segre, 2006).

This was the situation of early eukaryotic organisms, our ancestors, which came to use oxygen as an electron acceptor in oxidative metabolism biochemical networks, a process known as aerobic respiration (Henze and Martin, 2003; Taylor and McElwain, 2010). In principle, aerobic respiration can be understood as something similar to a reversal of the photosynthesis process, in which a carbon based substrate, usually glucose, is now broken down to produce energy for the organism. The eukaryotic capacity for aerobic metabolism is thought to have been acquired primarily through a symbiotic merger with the once free living proteobacteria that later developed into the mitochondrial organelle (Sagan, 1967). Current evidence from fossil records place this evolutionary event, which can be regarded as the birth of eukaryotic aerobic life, as having occurred ~2 billion years ago, nearly 1.45 billion years after the appearance of the early prokaryotes (Nisbet and Sleep, 2001). This evidence suggests that organisms capable of using oxygen for the generation of energy evolved in parallel to the changes in atmospheric oxygen concentrations during the Precambrian (Raymond and Segre, 2006; Berner et al., 2007; Harrison et al., 2010).

In the mitochondrial membrane, oxygen is used as a proton acceptor during the last stages of the respiratory chain. In brief, O_2_ in the mitochondrial matrix reacts with H^+^ ions creating H_2_O. This process maintains the powerful proton gradient between the mitochondrial matrix and the intermembrane space, which forces high energy protons through the ATP synthase molecule, thus fueling the production of ATP (Mitchell, 1961, 1979; Rich, 2003; Ward, 2006). The aerobic respiratory process wields ~30 molecules of ATP per metabolized glucose molecule, a 15-fold increase from the 2 ATP/glucose wield of the simple anaerobic glycolytic reaction (Rich, 2003). This massive increase in energy production efficiency was therefore a major evolutionary advantage for early eukaryotes.

The ability to use oxygen to generate energy was one of the main factors behind the emergence and diversification of complex life, including all Metazoans (Berner et al., 2007; Taylor and McElwain, 2010). There is evidence suggesting that the increase of atmospheric oxygen is linked to the Cambrian Explosion event which occurred ~500 million years ago, a period in which the paleontological record reveals that complex animal life evolved and diversified in an unprecedented manner (Ward, 2006; Fisher and Burggren, 2007; Taylor and McElwain, 2010) (Figure 1B). In addition, by producing the ozone layer and changing the overall composition of the atmosphere, the oxygenation of the atmosphere played an important role in the establishment of life in terrestrial habitats, thus vastly increasing the possibilities for ecological adaptations and biodiversity (Raven, 1997; Nisbet and Sleep, 2001).

## Transporting oxygen: the evolution of the cardiovascular and respiratory systems

The metabolic use of oxygen also represents one of the major evolutionary tradeoffs in the history of life on Earth: on one hand, eukaryotic organisms gained the ability to provide metabolic support to highly complex structures, such as the vertebrate brain; on the other hand, these organisms were now completely dependent on oxygen for their survival. Relatively simple organisms, including single cell eukaryotes and small metazoans, such as flatworms and nematodes, are capable of extracting their necessary supply of oxygen from the environment by direct diffusion (Fisher and Burggren, 2007). More complex organisms, however, need elaborate transport systems to ensure their needed share of O_2_ (Fisher and Burggren, 2007). This selective pressure likely favored the development of systems that provided the adequate supply of oxygen to each cell of the organism, even in the face of ever changing environmental conditions. Interestingly, different groups of animals developed distinct mechanisms for this task, each with its advantages and setbacks.

One the most interesting examples of the importance of the fine balance established in oxygen transportation systems occurs in the tracheal respiratory mechanism of insects. In this system, atmospheric oxygen diffuses directly to the cells of the animal through a mesh of fine tubular trachea and tracheoles that extend from the spiracles in the animal's exoskeleton to its deepest tissues (Wigglesworth, 1931). This system is extremely efficient when compared to indirect mechanisms of oxygen transport, where oxygen has to be diluted in bodily fluids and diffuse through many tissue interfaces. This is due to the fact that O_2_ diffusion is about 10 million times faster in air than in water, blood, and tissue (Hetz and Bradley, 2005). In the insect respiratory system, O_2_ only needs to diffuse across the water revested interface at the end of the tracheoles to reach the tissues. This allows insects to develop highly active lifestyles and exhibit behaviors with very high metabolic energy expenditure, such as flight (Lighton, 1996; Dudley, 1998). Considering that the tracheal respiratory system imposes a constriction on insect body size, as this system depends on a relatively direct diffusion between atmospheric air and tissues, large insects must have much longer trachea; the longer the trachea, the steeper is the oxygen gradient between the atmosphere and the deepest tissues of the organism (Harrison et al., 2010). This characteristic therefore limits the animal's capacity to maintain optimum oxygen levels in deep tissues, and is believed to have been, at least in part, responsible for the emergence and subsequent extinction of insect megafauna in the Carboniferous (when atmospheric O_2_ levels increased to around 30%) and Permian (when O_2_ levels went below 20%) periods, respectively (Dudley, 1998; Huey and Ward, 2005; Harrison et al., 2010) (Figure 1B). This would explain why all living insects are relatively very small when compared to their Carboniferous ancestors and modern vertebrates. This relationship between insect body size and atmospheric oxygen levels is, in our opinion, one of the most elegant examples of how environmental oxygen levels have directly influenced animal physiology and evolution.

In contrast to their insect relatives, vertebrates, including humans, rely on a different strategy: atmospheric oxygen is dissolved in blood, mainly bound to hemoglobin, and the heart pumps the O_2_ rich fluid to all cells in the body through a circulatory system. Therefore, vertebrates generally depend on complex respiratory systems, which provide an increased diffusion interface with atmospheric gasses, and a cardiovascular system, that transports oxygen to all cells of the body through the bloodstream (Taylor et al., 1999; Fisher and Burggren, 2007).

All vertebrates demonstrate some form of respiratory activity, meaning that oxygen absorbance is controlled by pumping environmental media through a respiratory diffusion interface, be it the coordinated propulsion of water through gills as seen in fish and amphibian larvae or the rhythmic pulmonary breathing of mammals (Taylor et al., 1999). The cardiovascular system also works continuously, through a constantly beating heart and reactive blood vessels, in order to maintain its primary function: guaranteeing optimum blood flow and oxygen supply (Taylor et al., 1999; Fisher and Burggren, 2007). Respiratory movements are dependent of central input from respiratory rhythm generating neural networks (Smith et al., 1991; Feldman and McCrimmon, 2008; Abdala et al., 2009b); likewise, cardiovascular parameters such as heart rate and peripheral resistance, although influenced by local metabolic and vascular physiological factors, are usually kept under tight control by the peripheral branches of the autonomic sympathetic nervous system, which in turn are also regulated by specific networks in the CNS (Guyenet, 2006).

In addition, almost all vertebrates, with the notable exception of the icefishes of the Channichthyidae family (Ruud, 1954; Sidell and O'Brien, 2006), rely on the chemical properties of hemoglobins, a class of heme-binding proteins with iron-bound porphyrin rings capable of reacting reversibly with oxygen. In vertebrates, these molecules largely increase the blood's O_2_ saturation capacity and, consequentially, the organism's oxygen transportation efficiency (Scholander, 1960; Hardison, 1996, 1998). Hemoglobins are actually a very ancient and versatile class of proteins that can be found in all major groups of living organisms, from bacteria and fungi to complex plants and animals, serving many different physiological functions, most of them related to oxygen transportation and homeostasis (Hardison, 1996, 1998). Vertebrate hemoglobins are mostly specialized for oxygen transportation from the lungs to the tissues and are extremely abundant in red blood cells (Hardison, 1998). In humans, for example, each gram of hemoglobin effectively binds to around 1.34 mL of O_2_ (Pittman, 2011). Considering that hemoglobin concentration is usually around 15 g per 100 mL of arterial blood, this translates into around 20.1 mL of hemoglobin-bound O_2_ per 100 mL of blood at full saturation. In the absence of this protein, i.e., if the system depended exclusively on diffusion, the same volume of blood would only be able to carry 0.3 mL of O_2_ (Pittman, 2011), which means that hemoglobin confers a ~70-fold increase in oxygen carrying efficiency.

The vertebrate paradigm of oxygen distribution has many advantages, as it allows for the existence of relatively large, complex, high energy demanding organisms (Fisher and Burggren, 2007). The diversification of vertebrates was also highly associated to the increase in atmospheric oxygen observed during the Carboniferous period, which, among other factors, probably contributed to the colonization of land by vertebrates and the transition from branchial to pulmonary respiration (Graham et al., 1995; Ward, 2006; Powell, 2010). Interestingly, during the reduction of atmospheric pO_2_ from ~31 to 21% in the Permian period which, as previously stated, very likely drove the insect megafauna to extinction, vertebrates are believed to have developed a series of adaptations that allowed them to increase their oxygen distribution efficiency, such as the parallel pulmonary circulation and the four chambered heart (Graham et al., 1997; Huey and Ward, 2005; Fisher and Burggren, 2007) (Figure 1B). Further adaptations in the cardiovascular and respiratory systems were also essential for the evolution of homeothermia in birds and mammals (Huey and Ward, 2005; Fisher and Burggren, 2007). Thus, the oxygen distribution system of vertebrates has very likely played a major role in the evolution of this group.

## The brain as a regulator of systemic oxygen supply: a major driving force in the evolution of the nervous system

Sensing external and internal oxygen levels and producing appropriate behavioral and physiological responses are part of the most preserved functions of the nervous system. All animals, including very primitive life forms such as jellyfish and nematodes, exhibit at least some form of behavioral response to reduced oxygen levels, which usually drives the organism to relocate to an environment with an adequate supply of O_2_ and produces physiological changes so as to maximize energy expenditure efficiency (Guyenet and Koshiya, 1995; Cheung et al., 2005; Hetz and Bradley, 2005; Thuesen et al., 2005; Fisher and Burggren, 2007). In animals with differentiated respiratory systems, such as insects and vertebrates, hypoxia not only evokes marked behavioral responses, but also produces changes in respiratory movements and autonomic adjustments in order to keep ideal blood flow and cellular oxygen supply (Holeton and Randall, 1967; Guyenet and Koshiya, 1995; Taylor et al., 1999; Hetz and Bradley, 2005; Vermehren et al., 2006; Sundin et al., 2007).

While some species, including diving animals such as turtles and whales, mountainous species like llamas, migratory birds and various species of fish have incredible tolerance to hypoxic environments (Lutz et al., 1980; Lutz and Milton, 2004; Nilsson and Lutz, 2004; Ramirez et al., 2007), most animals, including humans, experience severe tissue damage and death after minutes of hypoxia (Ramirez et al., 2007). Considering the evidence from less differentiated species and the importance of oxygen for maintaining complex life, it is highly probable that oxygen sensing and the coordination of behavioral adaptations to hypoxia were some of the very first roles of the nervous system. The need for an effective and integrative oxygen regulation mechanism has likely been one of the major driving forces in the evolution of the brain. This hypothesis makes even more sense when we consider that early life forms evolved in an aquatic environment, where O_2_ levels vary greatly in relation to variables such as depth, temperature and salinity (Weiss, 1970; Childress and Seibel, 1998; Sundin et al., 2007).

Therefore, the abilities of sensing environmental pO_2_ and producing effective homeostatic responses were likely to have endowed their early wielders with a selective advantage in relation to their peers that could not accurately coordinate their responses to hypoxia. This interpretation is further reinforced by recent discoveries on the role of O_2_-sensitive neurons in the nematode *C. elegans*, which show that oxygen sensing pathways deeply affect tonic and acute behavioral control and highlight the importance of this function both from an evolutionary and functional perspective (Busch et al., 2012).

**Figure 2:**
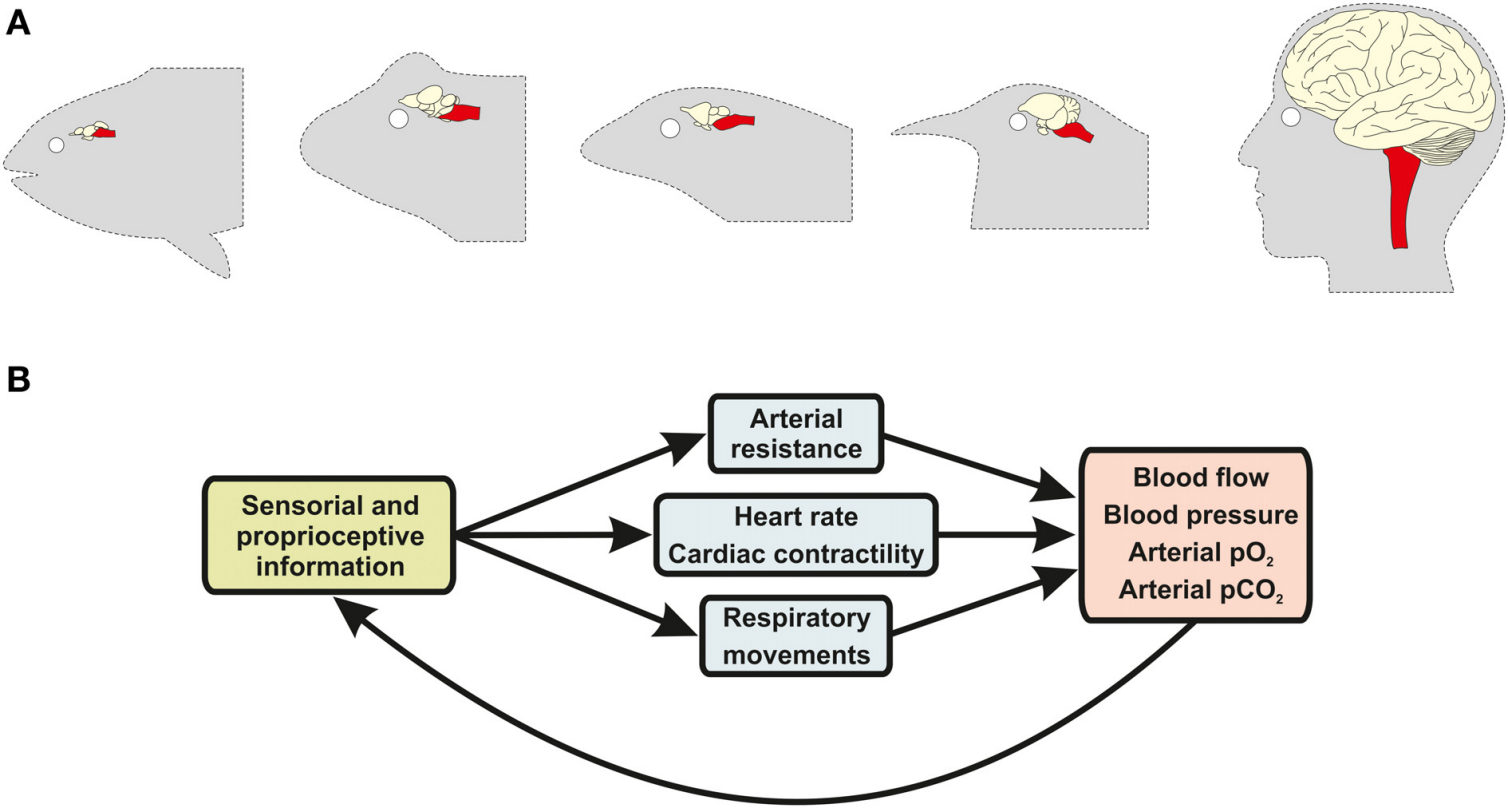
**Sensory-effector loops between the brain and the cardiovascular and respiratory systems regulate gas exchanges and blood flow. (A)** Schematic illustrations of the brain of animals from five major vertebrate taxa, including bony fish, amphibians, reptiles, birds, and mammals (ordered from left to right). Brainstem anatomy and structure (red highlight) has remained conserved across vertebrate evolution, despite major changes in other brain regions (non-highlighted), due to the fact that this brain structure performs similar functions in all vertebrate groups. **(B)** In all vertebrates, brainstem neurons receive information from specialized sensorial and proprioceptive organs, such as the carotid body glomus cells, and coordinate nerve activity outflow of the sympathetic and parasympathetic branches of the autonomic nervous system, as well as of respiratory motor nerves, inducing changes in respiratory movements (e.g., pulmonary breathing and opercular movements), blood pressure and cardiac output.

In vertebrates, the CNS constantly generates and regulates the neural activity of the cardiovascular and respiratory systems in response to intrinsic or extrinsic alterations, such as high levels of exercise, the sleep-wake cycle and, of course, environmental hypoxia (Taylor et al., 1999; Sundin et al., 2007). The respiratory system indeed cannot function without constant input from CNS networks that generate the rhythmic motor activity of breathing (Smith et al., 1991; Abdala et al., 2009b). In all vertebrates, the nervous system integrates proprioceptive signals and responds with coordinated vascular, cardiac and respiratory responses (Figure 2). In this context, the survival of the organism depends, at its very core, on the integrity of the brain, especially the brainstem nuclei that regulate breathing and autonomic control.

Highlighting the evolutionary importance of these regulatory neural functions is the fact that nearly all the major cardiovascular and respiratory control centers are located in the brainstem, one of the most primitive neuroanatomical region of the vertebrate nervous system (Northcutt, 2002). Lampreys and hagfish, the most primitive living species of the vertebrate lineage, already have anatomically and physiologically identifiable brainstem nuclei, which would place the development of this brain region at around 300 million years ago (Rovainen, 1985; Russell, 1986; Nishizawa et al., 1988; Ronan, 1988; Bardack, 1991). It is very likely that neural networks that regulate respiratory and cardiovascular function were amongst the first complex networks to develop during vertebrate brain evolution, as an efficient coordination of cardiorespiratory responses to hypoxia would have represented a major evolutionary advantage for early vertebrates.

## Moving from water to air: evolution of oxygen-sensitive peripheral chemoreflex

The term “chemoreflex” refers to a category of central autonomic and respiratory reflexes triggered by alterations in arterial blood levels of pCO_2_/pH and mainly pO_2_ and that result in the generation of adaptive and coordinated ventilatory and cardiovascular responses, as well in behavioral responses in order to move to an environment with safe oxygen levels. In this review we shall focus on the peripheral chemoreflex, which is primarily triggered by reductions in arterial pO_2_; recent reviews on the physiology and evolution of CO_2_/pH-dependent chemoreflexes can be found elsewhere (Sidell and O'Brien, 2006; Ainslie and Duffin, 2009; Dean and Nattie, 2010).

The peripheral chemoreflex has received great attention in the physiological sciences community ever since its Nobel Prize (1938) winning discovery by Corneille Heymans. We now know much about the mechanisms and central pathways involved in peripheral chemoreception. In this review we chose to look at this system from a different perspective; we will focus our discussions on the teleonomic motifs underlying the physiology of the peripheral chemoreflex in mammals. We wish therefore to dissect the adaptive roles of this biological system in an evolutionary context as deduced from its observable characteristics.

All studied vertebrates exhibit some form of specialized peripheral oxygen chemoreflex (De Burgh Daly, 1997; Milsom and Burleson, 2007; Sundin et al., 2007). In fish, the transduction of blood oxygen level signals is performed by neuroepithelial cells located in vessels positioned all across the respiratory passages, especially in the gills, the primary sites of oxygen absorption in most species (Milsom and Burleson, 2007; Sundin et al., 2007; Jonz and Nurse, 2012). Activation of the peripheral chemoreflex in fish produces reflex bradycardia, an increase in blood pressure, intense respiratory responses and a fleeing behavioral response, all features that are relatively conserved across the evolutionary timescale (Sundin et al., 2007). Neuroanatomical studies have shown that in fishes the sensory axons from chemoreceptors terminate in a brainstem area described as general visceral nucleus, or vagal sensory area (Kanwal and Caprio, 1987; Sundin et al., 2003a,b), which is involved in the modulation of the cardioventilatory responses to hypoxia and is homologous to the mammalian *nucleus tractus solitarius* (NTS) (Sundin et al., 2003a,b; Turesson and Sundin, 2003; Turesson et al., 2010).

In amphibians, peripheral chemoreceptor sites are seemingly homologous to those in fishes but with the anatomical and physiological reorganizations essential for the transition from aquatic to air breathing. For example, in larval amphibians the peripheral O_2_ chemoreception occurs in the gills, similarly to fishes, but over the course of typical metamorphosis, the first gill arch of the larvae develops to become the carotid labyrinth, which is the primary O_2_-sensing organ for the adult (Ishii and Oosaki, 1969; Milsom and Burleson, 2007; Jonz and Nurse, 2012). This vascular region is innervated by cranial nerve IX and the chemoreceptor cells located in it are one of the mediators of the rapid respiratory responses to hypoxia (Ishii and Oosaki, 1969; Milsom and Burleson, 2007).

As previously discussed, in aquatic habitats, O_2_ levels may vary greatly according to depth, temperature and other environmental factors (Weiss, 1970; Childress and Seibel, 1998; Sundin et al., 2007). Accordingly, peripheral chemoreflex pathways play a relatively greater role in setting baseline autonomic and respiratory parameters in aquatic animals in relation to mammals and other terrestrial vertebrates, as they are nearly constantly activated by fluctuations in water O_2_ concentration and are necessary to keep oxygen transport capacity at optimum levels (Sundin et al., 2007).

In lizards, turtles, birds and mammals there is a growing tendency for the concentration of chemoreceptor tissue in the main vessels leading from the lungs and into the brain, especially the carotid arteries and their homologs (Milsom and Burleson, 2007). This evolutionary trend is thought to be correlated with the transition from aquatic and bimodal breathing to exclusively aerial breathing and the transition from cardiovascular systems with intra-cardiac shunts to systems with fully isolated cardiac compartments (Milsom and Burleson, 2007), i.e., from a physiological situation in which environmental and blood levels of oxygen vary greatly to one in which these values are mostly kept at stable conditions.

Of course, as is always the case in evolutionary research, one cannot directly test the hypothesis that the variations in atmospheric O_2_ levels were the main driving force for the development of the cardiovascular and respiratory systems and the neural mechanisms for adaptation to hypoxia. All the arguments exposed so far have been based on correlations with fossil record and the analysis of the comparative anatomy and physiology of modern organisms. Nevertheless, one could identify which forms of experimental approaches and data would lend support to this hypothesis. One prediction of this hypothesis would be that animals with targeted dysfunctions in oxygen sensing mechanisms would have a lower reproductive success in face of hypoxic stressors simulating ancient atmospheric O_2_ levels. While to our knowledge no study has systematically tested these predictions in the context of reproductive success, we will demonstrate in the following sessions that there is a large body of experimental evidence confirming that the peripheral chemoreflex is indeed crucial for adaptive responses to hypoxic stressors similar to those faced by our early vertebrate ancestors.

There are also more direct ways of conducting experiments to test evolutionary hypotheses. One long term experimental study, conducted by Koch and Britton (2001, 2007), tackled the similar yet broader hypothesis that aerobic training capacity would be a determinant of evolutionary fitness. By artificially selecting animals with high and low intrinsic aerobic treadmill running capacity for multiple generations, they showed that selecting for low aerobic capacity lead to higher cardiovascular disease risk factors (e.g., high blood pressure, high free fatty acids, and visceral adiposity) and lower health factors (e.g., maximal oxygen consumption, endothelial nitric oxide production, and cardiac function) in relation to animals selected for high aerobic capacity. The question of how different environmental levels of O_2_ affect development and physiology of has also been addressed experimentally in insects and at least one species of vertebrate (Greenberg and Ar, 1996; Dudley, 1998; Peck and Maddrell, 2005; Owerkowicz et al., 2009; Harrison et al., 2010; Powell, 2010). In these experiments, animals are typically reared from embryonic life to adulthood under normoxic, hypoxic and hyperoxic conditions in relation to the current 21% atmospheric O_2_ percentage, and they have shown, for example, that insects raised in hypoxia had lower growth rates than their normoxic-raised counterparts (Greenberg and Ar, 1996; Peck and Maddrell, 2005; Harrison et al., 2010), and that alligators raised in hypoxic conditions show an adaptive enlargement of the heart and lungs (Owerkowicz et al., 2009), which provides experimental evidence for the hypotheses that environmental O_2_ concentration limits insect body size and that decreased levels of O_2_ are a strong pressure for developing bigger and more developed cardiovascular and respiratory systems, respectively. A very innovative study by Klok and Harrison (2009) went even further and studied the effects of hypoxia, normoxia and hyperoxia across multiple generations of *Drosophila* reproductive cycles, showing that indeed lower levels of oxygen favor the selection of flies with smaller body sizes. These experimental paradigms could be applied separately or in combination to test our hypothesis, for example by artificially selecting animals according to their intrinsic peripheral chemoreflex response sensitivity across multiple generations and testing whether that leads to systematic changes in reproductive success, life expectancy, disease incidence and general health factors, as well as if and how the results change when the animals are raised with different concentrations of environmental O_2_. However, it is important to note that these long term and sophisticated experiments remain to be performed.

## Physiology of the peripheral chemoreflex in mammals

**Figure 3:**
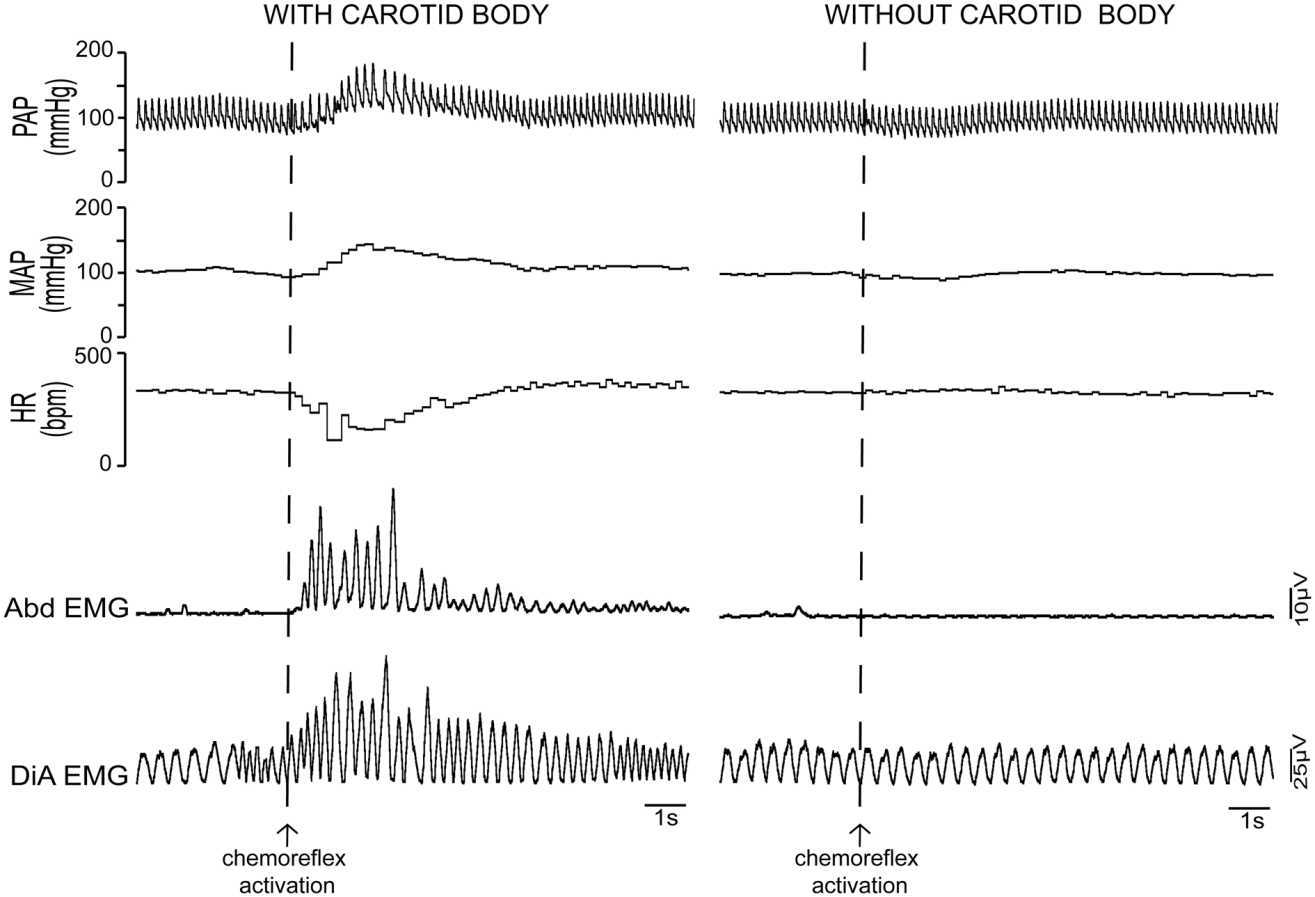
**Cardiovascular and respiratory responses to peripheral chemoreflex activation: importance of the carotid body**. Activation of the peripheral chemoreflex with environmental hypoxia in intact unanesthetized rats produces a marked increase in pulsatile (PAP) and mean arterial pressure (MAP), a decrease in heart rate (HR) and hyperventilation with forced inspiration and expiration, as can be seen by the strong changes in the eletromyographic (EMG) signals of the abdominal (Abd) and diaphragm (DiA) muscles. In rats that have been subjected to carotid body denervation, the hypertensive, bradycardic and respiratory responses to hypoxia are completely abolished. This fact highlights the essentiality of the peripheral chemoreflex pathway for the cardiorespiratory responses to systemic hypoxia.

In mammals, the peripheral chemoreflex is mediated mainly by clusters of specialized oxygen sensitive glomus cells localized in the carotid body and, to a lesser extent, in the aortic arch (Lahiri et al., 1981; Longhurst, 2008). These cells are thought to be homologous to the chemosensitive tissue of other vertebrates (Ishii and Oosaki, 1969; Milsom and Burleson, 2007). Glomus cells have an exponential sensitivity curve to blood oxygen levels (Lahiri et al., 1981; Feldman and McCrimmon, 2008), responding rapidly and intensely as the arterial pO_2_ decreases to less than 100 Torr. When stimulated by hypoxic conditions, carotid body glomus cells release excitatory neurotransmitters that excite adjacent terminals of the cell bodies located in the petrosal ganglia (Feldman and McCrimmon, 2008). Removal of the carotid body abolishes cardiorespiratory responses to hypoxia, highlighting the crucial role of this sensorial pathway (Figure 3). The physiology of peripheral chemoreceptor cells *per se* have been recently reviewed elsewhere (Kumar and Prabhakar, 2011). It is important to note that the cells can actually respond to a variety of chemical inputs, leading some authors to classify them as multimodal receptors (Kumar and Prabhakar, 2011). While this is a very interesting concept, both from an evolutionary and physiological point of view, the focus of this review is on the role of peripheral chemoreflex pathways in hypoxia and we will henceforth discuss this particular function.

Chemoreflex related neural information reaches the CNS via the glossopharyngeal nerve, and form their first synapses in the NTS, one of the major brainstem nuclei involved in cardiovascular and respiratory integration and control (Berger, 1979; De Burgh Daly, 1997; Longhurst, 2008; Accorsi-Mendonça et al., 2011). It should be noticed that the NTS is a heterogeneous nucleus, which processes multimodal sensory afferents, including gustatory, nociceptive and enteric inputs (Hill et al., 1983; Rinaman et al., 1989; Du and Zhou, 1990), as well as heterogeneous multimodal input from the carotid body (Kumar and Prabhakar, 2011). Recently, we demonstrated that NTS neurons receiving direct projections from the carotid body present a large variability in the latency of evoked glutamatergic currents, suggesting an intrinsic heterogeneity of the sensory pathways from the carotid body to the NTS (Accorsi-Mendonça et al., 2011; Accorsi-Mendonça and Machado, 2013).

**Figure 4:**
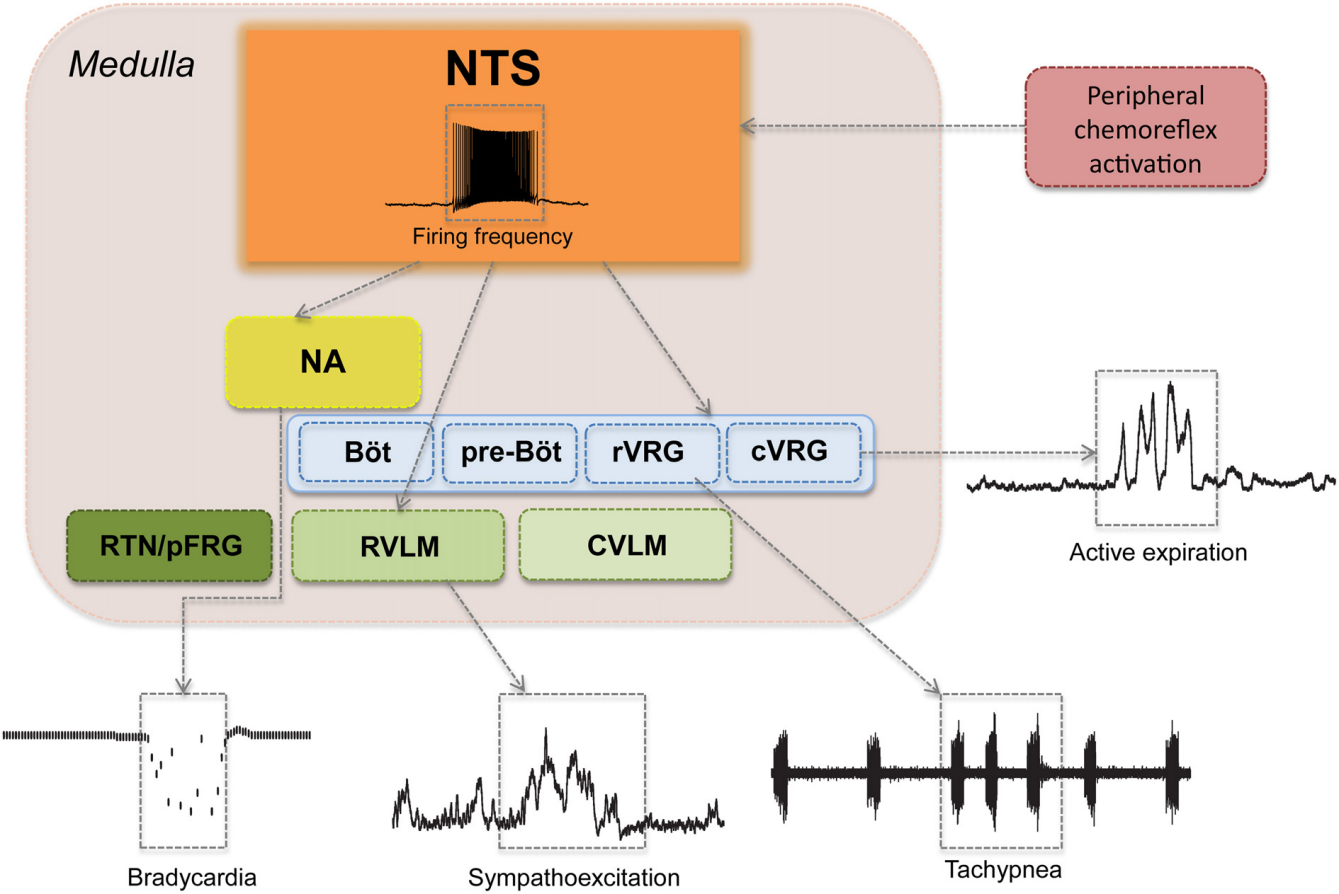
**Major pontine and medullary regions involved in the cardiovascular and respiratory responses to peripheral chemoreflex activation**. Aortic and carotid bodies and supra-medullary pathways are not shown. Observe that peripheral chemoreflex activation recruits several brainstem nuclei in series and in parallel in order to produce a patterned and integrated cardiorespiratory response to hypoxia, which is characterized by (1) bradycardia, represented here as a decrease in instantaneous heart rate; (2) sympathoexcitation, demonstrated here as increases in the amplitude of thoracic sympathetic nerve activity; (3) tachypnea, show as an increase in the frequency of phrenic nerve bursts; and (4) forced expiration, here seen as an increase in the activity of the abdominal muscles during the expiratory phase of the respiratory cycle. NTS, nucleus tractus solitarii; NA, nucleus ambiguous; RTN/pFRG, retrotrapezoid nucleus and the parafacial respiratory group; BötC, Bötzinger Complex; preBötC, pre-Bötzinger Complex; rVRG, rostral ventral respiratory group; cVRG, caudal ventral respiratory group; RVLM, rostral-ventrolateral medulla; CVLM, caudal-ventrolateral medulla.

The cardiovascular and respiratory responses to peripheral chemoreflex activation are mediated mainly by autonomic and respiratory nuclei of the brainstem (Figure 4) (Guyenet and Koshiya, 1995; Koshiya and Guyenet, 1996b; Machado et al., 1997; Costa-Silva et al., 2010; Moraes et al., 2011). The influence of higher regions of the forebrain and the midbrain, while crucial for the behavioral and emotional modulation of breathing and circulation, is not essential for the generation of sympathetic activity, eupneic respiratory rhythmogenesis nor the processing of the peripheral chemoreflex (Abdala et al., 2009b). Indeed, the cardiovascular and autonomic responses to carotid body stimulation persist virtually unchanged even in pre-collicularly decerebrate preparations of rats and mice (Braga et al., 2007a; Abdala et al., 2009b; Costa-Silva et al., 2010).

Neurons of the NTS integral to the chemoreflex pathway provide direct excitatory stimuli to the rostral ventro-lateral medulla (RVLM), a region rich in pre-sympathetic neurons and the main site of central sympathetic activity coordination (Guyenet and Koshiya, 1995; Koshiya and Guyenet, 1996a; Guyenet, 2000). RVLM pre-sympathetic neurons project directly to sympathetic neurons of the intermediate lateral (IML) column of the spinal cord and are responsible for the majority of the sympatho-excitatory component of the peripheral chemoreflex response, which causes the marked increases in peripheral vascular resistance, arterial blood pressure and cardiac inotropism following the activation of this reflex (Figures 3, 4) (Guyenet, 2000; Braga et al., 2007b; Longhurst, 2008). Another important nucleus that contributes to the sympathoexcitatory component of the peripheral chemoreflex is the pontine A5 area, which receives projections from the intermediate and caudal NTS and projects directly to the IML and other pre-sympathetic regions, such as the paraventricular nucleus of the hypothalamus (PVN) (Byrum and Guyenet, 1987; Guyenet and Koshiya, 1995; Guyenet, 2000). Putative chemoreflex-related NTS projections may also excite neurons of PVN (which in turn sends direct excitatory projections to the RVLM and the IML) and also contribute to the cardiovascular changes induced by carotid body stimulation (Olivan et al., 2001; Longhurst, 2008). In addition to these excitatory connections, there is recent evidence that the caudal-ventrolateral medulla (CVLM), an area known for its sympatho-inhibitory effects on the RVLM, may play a role in peripheral chemoreflex processing, mainly by regulating the intensity of the sympatho-excitatory response (Mandel and Schreihofer, 2009).

In rats, the peripheral chemoreflex pathways also involve a parasympathetic component, which produces intense bradycardia in situations of extreme hypoxia (Figures 3, 4) (Longhurst, 2008). The parasympathetic component of the peripheral chemoreflex is believed to be mediated mainly by projections of the NTS to the nucleus ambiguus (NA) (Nunez-Abades et al., 1990; Machado, 2001). This region contains pre-ganglionic parasympathetic neurons involved in cardiovascular regulation (Machado and Brody, 1988a,b). The role of this parasympathetic-mediated, and seemingly paradoxical, bradycardia is still poorly understood. One possibility is that the decrease in heart rate may actually protect the myocardium from hypoxic damage and physiological strain by limiting the increase of the heart's metabolic demand in face of a strong sympathetic stimulation (high cardiac contractility and increased peripheral resistance), but this hypothesis has not yet been proven. In humans, exposure to moderate hypoxia causes an increase in heart rate, which is thought to be consequence of the increase in sympathetic activity promoted by peripheral chemoreceptor activation (Houssiere et al., 2005).

The respiratory responses to peripheral chemoreflex activation include tachypnea and both forced inspiration and expiration (Figures 3, 4) (Paton et al., 1999; Braga et al., 2006; Abdala et al., 2009a; Granjeiro and Machado, 2009; Oliva et al., 2010; Moraes et al., 2011, 2012b). These responses are mediated mainly by direct projections of NTS neurons to the medullary ventral respiratory group (VRG), which includes the Pre-Bötzinger Complex (preBötC), the main central respiratory rhythm generator (Figure 4) (Nisbet and Sleep, 2001; Molkov et al., 2011). Other regions of the VRG, including the Bötzinger complex (BötC), the rostral ventral respiratory group (rVRG), and the caudal ventral respiratory group (cVRG), are also thought to be involved in the processing of the respiratory responses to chemoreflex activation, particularly in the forced inspiration and expiration pattern observed following carotid body activation (Guyenet, 2000; Abdala et al., 2009a; Mandel and Schreihofer, 2009; Zoccal et al., 2009b; Zoccal and Machado, 2011; Moraes et al., 2012b). The Retrotrapezoid nucleus (RTN) and the Parafacial Respiratory Group (RTN/pFRG), also receive chemoreflex related input (Figure 4) and are involved in the expiratory and sympatho-excitatory components of the response to hypoxia (Takakura et al., 2006; Abdala et al., 2009a; Abbott et al., 2011; Pagliardini et al., 2011; Takakura and Moreira, 2011; Damasceno et al., 2014a,b). In addition, there is growing evidence that NTS projections to pontine nuclei, especially to the parabrachial nucleus (PBN) and the Kölliker fuse (KF), also play an important role in the respiratory repercussions of chemoreflex activation (Abdala et al., 2009b; Costa-Silva et al., 2010; Song et al., 2011). Interestingly, it has been shown that in rats the sympathoexcitatory component of chemoreflex activation is strongly modulated by the respiratory pattern, with the peak of the sympathetic response occurring during expiration (Figure 4) (Koshiya and Guyenet, 1996a,b; Dick et al., 2004; Mandel and Schreihofer, 2009; Costa-Silva et al., 2010; Moraes et al., 2012b). While the precise pattern of entrainment between sympathetic and respiratory activity can vary widely between species, strain, recorded nerve and experimental preparation, this increase in sympathetic activity during expiration has been reported consistently in recordings of thoracic and splanchnic sympathetic nerves in both anesthetized rats and in *in situ* preparations (Dick et al., 2004; Mandel and Schreihofer, 2009; Costa-Silva et al., 2010; Moraes et al., 2012b). The physiological significance of this coupling is still not clearly defined, but current evidence suggest that it may improve blood delivery to the lungs and thus facilitate blood gas exchange and tissue perfusion, especially in conditions of metabolic challenges (Nilsson and Aalkjaer, 2003; Ben-Tal et al., 2012; Moraes et al., 2012a).

At rest, the peripheral chemoreceptor pathway contributes little to cardiovascular control in mammals; in baseline physiological conditions, baroreflex regulation of blood pressure and heart rate is predominant (Feldman and McCrimmon, 2008; Stickland et al., 2011; Mouradian et al., 2012). Carotid body denervation (CBD) can cause transient moderate hypoventilation in rats, but animals adapt after a few days (Mouradian et al., 2012). Due to the very sensitivity curve of the carotid body, the peripheral chemoreflex mechanism is activated mainly in situations of extreme hypoxic challenges, when the arterial pO2 is below 50 mmHg.

## Teleonomic function of the peripheral chemoreflex

We may now attempt to define the oxygen-sensitive pathway of the peripheral chemoreflex in terms of its teleonomic function, in light of the current physiological evidence. As previously discussed, this system is very ancient and evolved primarily as a mechanism to gauge environmental oxygen levels and produce constant autonomic, respiratory and behavioral responses (Milsom and Burleson, 2007; Sundin et al., 2007). The peripheral chemoreflex thus appears to have initially evolved as a mechanism to allow fish and amphibians to sense and respond to the highly variable environmental O_2_ concentrations in their watery habitat (Taylor et al., 1999; Milsom and Burleson, 2007; Sundin et al., 2007). In these animals, this reflex serves as an “environmental monitoring” system, constantly probing oxygen availability and producing appropriate adjustments (Taylor et al., 1999; Sundin et al., 2007).

In mammals, the distribution of peripheral oxygen sensing cells is concentrated in strategic arteries between the heart and the brain, mainly in the carotid bodies (De Burgh Daly, 1997). This allows these cells to monitor blood oxygen levels at the point of near maximum oxygenation and before any potentially deoxygenated blood actually arrives at the brain. In addition, the steep exponential sensitivity curve of the carotid glomus cells and the pronounced cardiorespiratory responses triggered by their activation reveal a system that evolved to respond to extreme situations, when intense responses are necessary for ensuring the life of the organism (Guyenet and Koshiya, 1995; De Burgh Daly, 1997; Machado, 2001). This is further confirmed by the minimal influence of peripheral chemoreceptor pathways for setting baseline cardiovascular and respiratory parameters in mammals (Feldman and McCrimmon, 2008). From an evolutionary point of view, this makes sense due to the relative stability of atmospheric oxygen levels.

Thus, in mammals the peripheral chemoreflex can be defined, in an evolutionary context, as a fundamentally preventive reflex; by initiating immediate adjustments to the sensing of hypoxic blood in the carotid arteries, the peripheral chemoreflex prevents the brain from being further exposed to low oxygen levels. It can be understood as an alarm system evolved to primarily protect the brain, and consequentially the rest of the organism, from the deleterious effects of hypoxia, which may include irreversible brain damage and even death.

But what kind of situations in the life of a mammal could cause hypoxia? While there is the possibility of intentional asphyxiation by a predator or competitor, it seems unlikely that the peripheral chemoreflex pathways could do much to help the organism in these situations. In light of the current literature, we believe that the peripheral chemoreflex is perhaps most important in specific pathological situations where systemic blood flow and oxygen absorption are severely impaired. This sensory pathway also contributes to the physiological adjustments to certain cardiorespiratory challenges, such as exercise and apnea diving. In the context of long-term maintenance of systemic homeostasis, the peripheral chemoreflex pathway is also crucial for altitude adaptation, a factor that has had huge impacts on human biogeography and health.

## The peripheral chemoreflex in health and disease: a hot-topic in integrative and translational physiological research

It has been shown that several different physiological challenges in humans and other mammals involve the activation of peripheral chemoreflex neural pathways. Perhaps the clearest example is that of ventilatory acclimatization to high altitude, which is characterized by a marked increase in ventilation when in situations of prolonged hypoxia. In experimental animals, prolonged exposure to normobaric or hypobaric hypoxia induces an increase in carotid body activity and sensitivity that parallels the ventilatory adaptations (Barnard et al., 1987; Vizek et al., 1987; Nielsen et al., 1988). In addition, CBD surgery severely reduces the ventilatory response to sustained hypoxia in experimental animals and human patients, which implies that the carotid body plays a crucial role in altitude acclimatization (Forster et al., 1981; Roeggla et al., 1995; Bisgard, 2000). Interestingly, continued exposure to hypoxia can actually reduce carotid chemoreflex sensitivity by both central and peripheral mechanisms (Bascom et al., 1990; Tatsumi et al., 1991, 1995), an effect that could be related to homeostatic plasticity mechanisms (Turrigiano and Nelson, 2000). It should be noted that high altitudes imply levels of available atmospheric O_2_ ranging from 90% to around 45% of the atmospheric O_2_ at sea level (corresponding to around 19% to 10% of isobaric O_2_ percentage) (Baillie, 2010), which would be similar to the troughs in atmospheric O_2_ levels estimated for the Devonian, late Permian/early Triassic and Jurassic periods (Figure 1). While we cannot test whether the peripheral chemoreflex was indeed a crucial mechanism for adaptation to ancient O_2_ concentrations, the fact that it is essential for the appropriate acclimatization to hypoxia in modern mammals (Roeggla et al., 1995) suggests that this might have been the case.

There is a large body of evidence that exercise can recruit peripheral chemoreflex pathways, and that this activation is important for exercise hyperpnea (Wasserman et al., 1975; Whipp, 1994; Croix et al., 1996; Whipp and Ward, 1998; Fukuoka et al., 2003; Stickland et al., 2011). Studies in humans suggest that peripheral chemoreflex drive can contribute from ~15% of the exercise ventilatory response, in moderate exercise under normoxic conditions (Croix et al., 1996) to over 50% of this response, in intense exercise under hypoxic conditions (Wasserman et al., 1975; Whipp, 1994; Whipp and Ward, 1998; Fukuoka et al., 2003). Studies by Stulbarg et al. (1989) reported that patients with carotid body resection exhibit a decreased ventilatory response and experience severe hypoxemia during maximal exercise, suggesting that the peripheral chemoreceptor pathways play a crucial role in the respiratory adaptation to this challenge. Likewise, peripheral chemoreflex pathways play important roles in the cardiovascular responses to exercise, including the increase in muscular blood flow and heart rate, which are common during exercise conditions (Stickland et al., 2011).

In the last few decades, several studies revealed that the peripheral chemoreflex is also involved in the adaptation to several pathological conditions. There is a very large body of evidence pointing to a crucial role for the carotid chemoreflex in the progression of the pathophysiology of obstructive sleep apnea (OSA). This disease is characterized by a loss of upper airway muscle tonus during sleep, which causes obstruction of respiratory airflow leading to hypoxia and, consequently, activation of the peripheral chemoreflex, including an increase in heart rate, arterial pressure and respiratory effort (Somers et al., 1995; Narkiewicz and Somers, 1997, 1999, 2001; Narkiewicz et al., 1998a,b; Fletcher, 2001; Usui et al., 2005; Serebrovskaya et al., 2008). OSA affects ~24% of middle aged men and 9% of middle aged women (Wolk et al., 2003b); it is strongly associated with obesity, hypertension and elevated sympathetic activity even during wakefulness (Carlson et al., 1993; Narkiewicz and Somers, 1997; Wolk and Somers, 2003; Wolk et al., 2003a,b; Spaak et al., 2005). It is believed that OSA can have a causal role in the development of hypertension (Narkiewicz and Somers, 1999). Patients with OSA exhibit increased peripheral chemoreflex sensitivity, which is paralleled by an increase in sympathetic activity and the development of neurogenic hypertension (Narkiewicz et al., 1998c, 1999, 2005; Narkiewicz and Somers, 1999). The mechanisms by which this pathological condition generates these physiological effects are, however, poorly understood.

The development of animal models of OSA has proven to be a tricky problem. Over the last two decades, one of the most promising models for this disease has been the use of chronic intermittent hypoxia (CIH) protocols. This experimental paradigm consists of exposing animals, usually rats, to varying levels of environmental pO_2_. Typically, pO_2_ periodically changes from normoxic levels (21%) to extreme hypoxia (≈5%) for a few seconds; this is repeated many times during at least an 8 h time period for various days (Fletcher et al., 1992a; Fletcher, 1995, 2000; Fletcher and Bao, 1996a,b; Zoccal et al., 2008, 2009a,b; Zoccal and Machado, 2010, 2011). However, this method is far from being an accurate model of the pathological stimuli of OSA, given that it does not reproduce the transient hypercapnia nor the physical obstruction of the upper airways observed in human OSA patients. However, since it produces consistent intermittent hypoxia, it is well accepted as an experimental model of sleep apnea. Novel methods, based on actual airway obstruction, have recently been developed and they may prove to be very useful for unraveling the full pathophysiology of OSA (Farre et al., 2007; Nacher et al., 2007; Schoorlemmer et al., 2011).

Exposure to CIH does mimic one aspect of OSA, i.e., recurrent peripheral chemoreflex activation, and also causes hypertension and sympathetic over-activity in rats (Fletcher et al., 1992a,b,c; Fletcher, 1995, 2000, 2001; Fletcher and Bao, 1996a,b; Bao et al., 1997a,b), which validates, to a certain point, the extrapolation of concepts and discoveries based on the physiological effects of CIH exposure in experimental animals to the clinical conditions of OSA patients. Rats exposed to CIH present elevated arterial blood pressure (Zoccal et al., 2007, 2009a), an increase in the neural, respiratory and autonomic components of peripheral chemoreflex activation (Peng et al., 2001, 2003; Rey et al., 2004; Braga et al., 2006), active expiration even at rest (Zoccal et al., 2008, 2009b; Zoccal and Machado, 2010, 2011) and, most interestingly, an increase in sympathetic activity that correlates in time with the active expiratory activity (Zoccal et al., 2008, 2009b; Zoccal and Machado, 2010, 2011; Moraes et al., 2012a, 2013) suggesting a prolonged alteration in the synaptic determinants of the neuronal network controlling sympathetic-respiratory coupling (Moraes et al., 2013). Most importantly, these physiological effects of CIH depend upon the integrity of the peripheral chemoreflex pathway in order to be established, but removal of the CB after exposure to CIH by itself does not reverse the changes in sympathetic-respiratory coupling (Fletcher et al., 1992a; Lesske et al., 1997; Fletcher, 2001; Zoccal et al., 2008).

In addition, CIH exposure is known to have profound effects in glutamatergic synaptic processing in the NTS (Kline et al., 2007; Kline, 2010; Costa-Silva et al., 2012), including a reduction in evoked excitatory post-synaptic current amplitude on second order NTS neurons, indicating the existence of homeostatic synaptic plasticity mechanisms that may counteract the drastic increase in afferent carotid body information. The mechanisms of these changes have been elucidated recently, and have been shown to be dependent on a reduction in the number of active synapses in the NTS (Almado et al., 2012). Taken together, the current evidence show that CIH exposure, and thus probably OSA, result in profound alterations of the ponto-medullary neural networks that regulate respiratory activity, which seems to be the cause of the increase in the sympathetic activity, considering that electrophysiological properties of RVLM pre-sympathetic neurons are not altered in rats submitted to CIH (Moraes et al., 2013). These can be interpreted as the fundamental origins of cardiovascular risk in OSA patients.

Interestingly, we have recently shown that similar adaptive processes, including changes in respiratory-sympathetic coupling induced by a synaptically driven alteration of the ponto-medullary respiratory network, are also involved in the acclimatization to sustained hypoxia (Moraes et al., 2014), suggesting that this form of network reorganization might represent a general motif—potentially triggered by the peripheral chemoreflex—for adaptation to every form of environmental hypoxia.

Another pathological condition in which the peripheral chemoreflex plays a major role is congestive heart failure (CHF), which is defined as the inability of the heart to provide appropriate blood supply to all regions of the body and it can be caused by several factors, including hypertension, myocardial infarction and parasitic infections such as Chagas' Disease (McMurray and Pfeffer, 2005; Parra et al., 2012). It is estimated that 1 in 5 people are at risk of developing CHF until the age of 40 (McMurray and Pfeffer, 2005; Roger et al., 2011). This pathological condition is extremely debilitating and deadly: around 30–40% patients die within the first year after CHF diagnosis and 60–70% die within 5 years after this event (McMurray and Pfeffer, 2005). CHF is accompanied by a persistent sympathetic over-activation; this factor has a “double-edged” effect on cardiac function in CHF patients: on one hand the increased sympathetic activity adjusts heart rate and cardiac contractility to keep optimum blood flow in spite of myocardium tissue damage; on the other hand this prolonged sympathetic over-activation has several deleterious effects, such as arrhythmias and increased myocardial cell death, which ultimately increase the risk of sudden death (Chugh et al., 1996; Esler et al., 1997; McMurray and Pfeffer, 2005).

There is a large body of evidence linking peripheral chemoreflex potentiation with the sympathetic over-activation observed in CHF (Chua et al., 1997a,b; Ponikowski et al., 1997; Schultz and Sun, 2000; Ding et al., 2011; Guimarães et al., 2011). In human patients, augmented peripheral chemoreflex sensitivity is significantly associated with an increase severity of CHF symptoms, including sympathetic over-activity and a decrease in baroreflex sensitivity (Chua et al., 1997b; Ponikowski et al., 1997). Conversely, transient inactivation of peripheral chemoreceptors with hyperoxia increases the low-frequency and high frequency components of heart rate variability and improves baroreflex sensitivity (Ponikowski et al., 1997). In experimental animal models of CHF, there is also an enhancement of peripheral chemoreflex function associated to elevated sympathetic tonus (Sun et al., 1999a,b). This potentiation of the peripheral chemoreflex involves both changes in carotid body sensitivity and in the central processing of chemoreceptor input, as well as interactions with other autonomic reflexes such as the cardiac sympathetic afferent reflex (Sun et al., 1999a; Li and Schultz, 2006; Li et al., 2006; Reddy et al., 2007; Wang et al., 2008; Ding et al., 2011).

Given that the mammalian peripheral chemoreflex can be understood as an “alarm” system, adapted to respond mostly to extreme hypoxic challenges, it seems logical, in hindsight, that it plays such important roles in human pathologies that strain the cardiovascular and respiratory systems to their limits. This adds a new layer to the interpretation of the evolutionary importance of this sensory pathway: with the exception of animals which experience diving and apnea as part of their life history, the peripheral chemoreflex responds primarily to diseases and challenges that affect oxygen transport efficiency, including exercise, altitude acclimatization, upper airway obstructions and heart failure.

In the case of CHF, for example, the overactivation of the peripheral chemoreflex is a situation in which a complex regulatory system is operating under extreme conditions, in which the structure (heart) is falling but the reflex system processed by the brainstem is working as expected to compensate the lack of proper oxygen level in the entire system and especially to the brain. The lack of an appropriate response of the heart leads to a pathological overactivation of the sympathetic system, which then contributes to the progression and symptomatology of the disease. This contrasts with the healthy adaptations to high altitudes and exercise, where both the peripheral chemoreflex pathways and the cardiovascular and respiratory systems are working together to respond to an environmental or physiological stressor. When the peripheral chemoreflex, a neural system whose primary function is as an acute alarm mechanism, is activated chronically due to defects in the structure of the respiratory (as in OSA) or cardiovascular (as in CHF) systems, there is an overactivation of sympathetic neurotransmission that can exacerbate the pathophysiological consequences of the systemic diseases, instead of compensating for them. Therefore, mechanical dysfunctions in the heart or in the resistance of the upper airways can affect the sensory and reflex neural regulatory mechanisms due to an open-looping process, which makes the original pathophysiological condition even worse. In other words, problems in the “hardware” (e.g., the heart) will make the “software” (sensory and neural pathways of the chemoreflex) work against the integrity of the overall system, since it has not evolved to work in an adverse scenario in which the structural components of the system are failing. This is a clear example of how a mechanism that is highly adaptive in face of certain physiological stressors (such as altitude and exercise) can become maladaptive in chronic heart and respiratory diseases.

The recent findings of peripheral chemoreflex participation in generating sympathetic over-activity in the aforementioned diseases seem to have consolidated this pathway as a hot-topic in cardiovascular and respiratory physiology and pathophysiology. But can this newfound knowledge be translated into treatments for human patients? And if so, how?

One exciting new avenue of research guided by our understanding of the peripheral chemoreflex system is the surgical removal of the carotid body as a viable therapeutic approach to treating sympathetic overactivation in diseases such as severe resistant hypertension and CHF (McBryde et al., 2013; Niewinski et al., 2013; Paton et al., 2013a,b). This strategy stems, of course, from the recognition of this system as the physiological origin of pathological sympathetic overactivity in various cardiovascular diseases, as we discussed in this review. While this is an invasive and potentially dangerous procedure, it could prove an interesting clinical approach in the future. The success of these therapies would also be an experimental demonstration that plasticity of the peripheral chemoreflex pathway in CHF is indeed maladaptive—in contrast to the adaptive processes observed in physiological conditions—and that identifying the mechanisms of carotid chemoreflex adaptation can provide potential therapeutic targets for cardiovascular and respiratory diseases.

Recent findings have pointed out that certain protocols of volitional control of breathing, including those traditionally practiced in Yoga, can change the sensibility of the peripheral chemoreflex in humans (Spicuzza et al., 2000; Bernardi et al., 2001; Pinheiro et al., 2007) and even reduce arterial blood pressure in patients with essential hypertension (Pinheiro et al., 2007). The fundamental advantage of humans in the evolutionary landscape is the range of our behavioral adaptations, especially those guided by an evidence-based interpretation of the world. Increasing our understanding of the neuroplastic phenomena behind chemoreflex long-term adaptations might also allow us to design breathing exercises (a form of behavioral adaptation) that will specifically target peripheral chemoreflex function in order to improve cardiorespiratory function and quality of life in patients with heart failure, hypertension and other diseases.

In summary, we have seen that neural mechanisms of oxygen sensing in general, and the peripheral chemoreflex in particular, have played a pivotal role in the evolution of life. Given the importance of oxygen sensing pathways to homeostasis, dysfunctions in the peripheral chemoreflex result in disease states and severely limit the response to environmental challenges in both humans and experimental animal models. As cardiovascular and respiratory diseases continue to be one of the main causes of death in the world, research on how the peripheral chemoreflex functions in health and disease, and on how we can manipulate this pathway for therapeutic purposes, is more promising and necessary than ever.

## Author contributions

Benedito H. Machado had the initial insight for developing the manuscript. Kauê M. Costa and Benedito H. Machado executed the majority of the literature review and wrote the main body of the manuscript. Davi J. A. Moraes and Daniela Accorsi-Mendonça contributed with figure construction, wrote sections of the text and critically revised the manuscript.

### Conflict of interest statement

The authors declare that the research was conducted in the absence of any commercial or financial relationships that could be construed as a potential conflict of interest.
